# Molecular Origins of Nonfrozen Water in Polyelectrolyte Brushes

**DOI:** 10.1021/acs.langmuir.5c06138

**Published:** 2026-03-06

**Authors:** George Mallinos, Md. Golam Kibria, Saveen Jayaweera, Ali Dhinojwala

**Affiliations:** School of Polymer Science and Engineering, 1076 The University of Akron, Akron, Ohio 44325, United States

## Abstract

Polyelectrolyte brushes (PEBs) are promising coatings for reducing ice adhesion and regulating water freezing at interfaces, yet direct measurements of nonfrozen water retention at subzero temperatures remain scarce. Here, we investigate the freezing behavior of water confined in poly­([2-(methacryloyloxy)­ethyl]­trimethylammonium) (PMETA) brushes with chloride, iodide, and sulfate counterions using a custom-built low-temperature attenuated total reflectance infrared spectroscopy system. Furthermore, we quantify the fraction of water that was present within the brush that does not freeze as well as the changes in polymer volume fraction within the brush as a function of temperature. Spectroscopic analysis of water vibrational modes reveals that PMETA brushes retain 25–35 vol. % water even at −60 °C, providing direct evidence of substantial water confinement in charged polymer networks. These findings advance the fundamental understanding of interfacial water behavior in PEBs and suggest molecular design strategies for engineering anti-icing and cryo-lubricating surface coatings.

## Graphical abstract

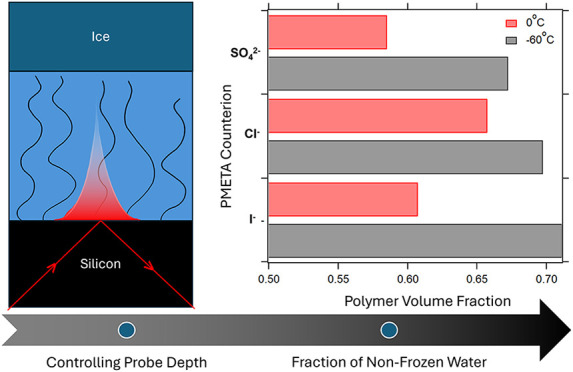

## Introduction

Understanding the fraction of water that remains unfrozen at low temperatures is critical across a wide range of scientific and technological fields. In biological systems, the extent of water freezing directly influences cell viability, tissue preservation, and freeze tolerance in organisms. One example is the ability of wood frogs (*Rana sylvatica*) to produce glucose to prevent their bodies from freezing.^1–4^ It was determined that 65.4% of the total body water was frozen;^2,5,6^ in contrast, the water frog (*Rana ridibunda*) is unable to survive above 58% freezable water at a temperature of −3 °C.^7^

In food science, the frozen water fraction governs ice crystal formation, which affects the texture, stability, and shelf life of frozen products.^8,9^ In 2018, Wang et al. used differential scanning calorimetry (DSC) on different enzyme-treated dough and found that samples with cellulose had reduced the amount of freezable water compared to controls by 75%, allowing the frozen dough to stay fresher for longer.^10^ Pradipasena et al. studied solutions of sugars and starches (glucose, dextran, potato starch) and evaluated them for fraction of frozen water vs temperature, revealing that higher molar mass increases freezable water content and helps predict freezing behavior in food systems.^8^ In 1997, a DSC study on beef muscle quantified nonfreezable water ranging from 49.18 to 29.69% for temperatures ranging from −5 to −65 °C to aid in better understanding how different freezing temperatures can affect meat in terms of appearance, texture, taste and packaging requirements.^11^

In materials science and civil engineering, frozen water content alters thermal conductivity, mechanical integrity, and freeze–thaw durability of porous materials such as hydrogels or cement-based composites.^12^ Despite the diversity of applications, a unifying principle is that the frozen fraction of water—not just the total water content—serves as a functional determinant of structural and phase behavior in complex aqueous systems. Accurate quantification of this frozen fraction, often via DSC or spectroscopic techniques such as Raman spectroscopy, provides essential insight for optimizing system performance, predicting failure modes, and designing formulations with improved freeze–thaw resilience.^13^

This ability to calculate the amount of nonfrozen water is also important in the anti-ice field, where unwanted ice accumulation poses significant challenges across a wide range of applications in the aerospace field,^14^ power transmission,^15^ food preservation,^16^ and transportation infrastructure.^17^ One promising strategy to mitigate ice adhesion and increase the amount of nonfreezable water involves the use of polyelectrolyte brushes (PEBs), in which polymer chains are grafted to surfaces that carry charged functional groups. Over the past several years, numerous studies have demonstrated the ability of PEBs to reduce ice adhesion;^18–23^ however, the underlying amount of freezable water within the swollen brush network has been difficult to quantify.

In 2019, Liang et al. demonstrated the ability of polyelectrolyte brushes to significantly reduce ice adhesion. DSC was used to estimate the amount of nonfreezable water in alginate–poly­[2-(methacryloyloxy)­ethyl]­trimethylammonium chloride (PMETAC) solutions as a proxy for PEB by incrementally increasing the water content in polymer samples until a melting endotherm corresponding to ice formation was detected. The onset of this melting peak was used to define the threshold at which freezable water was present; water added below this threshold was considered nonfreezable. Using this approach, it was determined that approximately 60% of the mass of polymer was considered nonfreezable.^19^ We recently reported a phase diagram of a PMETAC solution in contact with ice phase.^24^ While these studies may be useful for understanding bulk PMETAC systems, these systems may not be similar to those for PEBs in contact with water/ice.

More recently, in 2024, Biro et al. employed a custom-built total internal reflection Raman spectrometer to directly probe the frozen and nonfrozen water fractions within a cross-linked polyelectrolyte film.^18^ By systematically varying the incident angle of the Raman excitation beam, they controlled the penetration depth of the evanescent field, enabling depth-resolved measurements both within and beyond the polyelectrolyte film. By employing control Raman spectra of water, polymer, and ice, the experimental spectra were deconvoluted to quantify the relative contributions of polymer, nonfrozen water, and ice. Since the Raman spectrum collected at the low temperature was used as the reference of 100% ice, the results were already biased to show that all water within the cross-linked polyelectrolyte films is expelled from the film and freezes at low temperatures.

In 2025, Tamamoto et al. used Fourier-transform infrared (FTIR) spectroscopy to investigate the swelling behavior of nonionic, hydrophilic polymer brushes in response to localized water exposure at low temperatures.^21^ Since these experiments were conducted in transmission mode, the edge of a water droplet in contact with the brush layer was used to reduce the contribution of bulk water signals in the IR spectra. The transmission IR data revealed that during freezing of the precursor water film, the polymer brushes underwent deswelling—presumably to accommodate the outward flux of water molecules as they joined the growing ice phase. This observation led to the conclusion that a fraction of nonfrozen water remained confined within the brush network after ice formation. To further quantify this residual water, polymer brushes were grown in a DSC pan and were used to estimate the fraction of nonfrozen water in the polymer brush, which was approximately 0.5.

Although the studies discussed above provide approaches for estimating the fraction of nonfrozen water within the brush, none reports a direct measurement of this quantity. It is important to note that we define the fraction of nonfrozen water as the fraction of original water content that remains unfrozen. Previous reports seeking to calculate the overall water content of polyelectrolytes have primarily relied on quartz crystal microbalance;^25–27^ however, this technique is not suitable at subzero temperatures and cannot distinguish between liquid water and ice. Several studies have employed various techniques to investigate water behavior in polyelectrolyte multilayer (PEM) and PEB systems. For example, FTIR has been utilized to examine the O–H stretching region in PEMs, providing insights into swelling behavior and the spatial distribution of water molecules around ion pairs.^28^ Additionally, techniques such as neutron reflectivity^29^ and combined ellipsometry–quartz crystal microbalance (QCM) measurements^30^ have been used to quantify water uptake in both PEBs and PEMs. Despite these advancements, no established method currently exists to characterize the fraction of nonfrozen water in PEBs as a function of temperature.

Here, we present an approach to quantify the nonfrozen fraction of water within polyelectrolyte brushes using a custom-built, low-temperature, multibounce attenuated total reflection infrared (ATR-IR) spectroscopic setup. This approach enables in situ, surface-sensitive measurement of brush-confined water during freezing, overcoming the limitations of bulk techniques such as DSC or NMR. Unlike previous studies that infer hydration water indirectly through ice adhesion or swollen-state analysis,^18,21^ the custom low-temperature ATR-IR chamber directly probes nonfrozen water within the brush relative to surrounding ice. We investigated a positively charged polyelectrolyte, poly­[2-(methacryloyloxy)­ethyl trimethylammonium] (PMETA), over a temperature range from 0 to −60 °C, employing three different counterions: Cl^–^, I^–^, and SO_4_^2–^. Our counterions were chosen based on the Hofmeister series, from which we chose a weak monovalent kosmotrope (Cl^–^), a strong monovalent chaotrope (I^–^), and a strong divalent kosmotrope (SO_4_^2–^). Our results reveal that relative to the amount of water present in the swollen brush at 0 °C, 83% of water remains unfrozen for Cl^–^ counterions as compared to 63 and 69% for I^–^ and SO_4_^2–^, respectively. In addition, the polymer volume fraction was calculated, and the phase behavior was modeled using a modified Flory–Huggins framework with one variable, grafting density, while taking the degree of counterion dissociation to be equal to 1. By fitting the experimental data to this theoretical model, the effect of grafting density on the phase behavior was mapped. These results represent a direct measurement of liquid water within polyelectrolyte brush networks at subzero temperatures as well as the quantification of polymer volume fraction using IR spectroscopy.

## Experimental Section

### Substrate Preparation and Cleaning

Polyelectrolyte brushes were grown on multibounce silicon ATR-IR crystals (PIKE Technologies). Prior to functionalization, the substrates were cleaned using a piranha solution composed of 3 parts 30% hydrogen peroxide (H_2_O_2_, Sigma-Aldrich) and 7 parts 12 M sulfuric acid (H_2_SO_4_, Sigma-Aldrich). The substrates were immersed in piranha solution for 30 min, followed by thorough rinsing with ultrapure water (Millipore, pH 6–7). After rinsing, the substrates were dried under a stream of high-purity nitrogen and subjected to plasma treatment for 5 min to enhance surface hydroxylation immediately prior to initiator deposition.

### Initiator Functionalization

Vapor-phase initiator deposition was conducted in a 1-L glass vacuum desiccator free of grease to avoid contamination. The ATR-IR crystal was only initiated on the top surface to prevent PEB signals from other surfaces. Inside the desiccator, a small glass beaker containing 5 μL of 3-(triethoxysilyl)­propyl 2-bromo-2-methylpropanoate (93%, TCI Chemicals) served as the initiator source. The system was evacuated to a pressure of 140 mTorr using a mechanical vacuum pump before being sealed. The desiccator was placed in an oven at 80 °C for 4 h to promote covalent attachment of the initiator onto the plasma-activated silicon oxide surface through a vapor-phase silanization reaction. Following initiator deposition, the substrates were sequentially sonicated in toluene (Fisher Scientific, 99.5%) for 2 min to remove unwanted residues. The substrates were again dried under nitrogen prior to polymerization. Silane-based ATRP initiators form well-defined monolayers on oxide substrates via vapor-phase silanization, as confirmed by XPS, ellipsometry, and contact-angle measurements. In addition, the resulting initiator density directly determines polymer brush grafting density and conformation.^31–33^

### Polymer Brush Synthesis via ARGET-ATRP

Surface-initiated polymerization of poly­([2-(methacryloyloxy)­ethyl]­trimethylammonium chloride) (PMETAC) brushes was performed using activators regenerated by electron transfer atom transfer radical polymerization (ARGET-ATRP).^34^ In a typical reaction, 5.52 g of [2-(methacryloyloxy)­ethyl]­trimethylammonium chloride (METAC; 75 wt % in H2O, Sigma-Aldrich, 26.6 mmol), 0.93 mg of CuBr_2_ (Sigma-Aldrich, 4.16 μmol), 6.63 mg of 2,2′-bipyridyl (bipy, >99%, Sigma-Aldrich, 42.5 μmol), and 7.45 mg of ascorbic acid (Fisher Scientific, 42.5 μmol) were dissolved in 5 mL of a 1:1 (v/v) methanol/water mixture under gentle nitrogen flow and purged for 10 min. Separately, initiator-modified substrates were placed in a sealed reaction vessel and were purged with nitrogen for 10 min.

The deoxygenated reaction solution was then transferred to the reaction vessel containing the substrate via a gastight glass syringe. The sealed vessel was maintained at room temperature for 1 h to allow polymer brush growth. After polymerization, the substrates were thoroughly rinsed with multiple changes of methanol and ultrapure water, followed by sonication in methanol and water for 5 min each. The resulting PMETAC brush thickness was approximately short, as determined by ellipsometry (measured under ambient relative humidity of 20%).

### Counterion Exchange Procedure

To exchange the counterions of the grafted PMETAC brushes, silicon crystals with brushes grown on the top surface were placed in a 150 mL beaker. The beaker was then filled with a 100-mM aqueous solution of a sodium or potassium salt containing the desired counterion. The solution was stirred for 90 min to allow for efficient ion exchange. After treatment, the samples were thoroughly rinsed with excess Milli-Q water and were subsequently dried under nitrogen. X-ray photoelectron spectroscopy (XPS) results of the counterion exchange can be found in Figure S6.

### Weighted Average Calculation

To quantify temperature-dependent changes in the OH stretching region (3100–3600 cm^–1^), we calculated the weighted average wavenumber by integrating the product of wavenumber (ν) and absorbance (*A*) and normalizing by the integrated absorbance (eq 1).

1 $$w\mathrm{eighted}\;a\mathrm{verage}=\frac{\sum \nu A}{\sum A}$$

### Thermodynamic Model for Equilibrium of Polyelectrolyte Brush in Contact with Ice

In this section, we develop the conditions for equilibrium between the PE brush in contact with ice. This thermodynamic model can be used to calculate the volume fractions of nonfrozen water within the PE brush. For the coexistence of the ice phase in equilibrium with the PEB, we equate the chemical potentials of water molecules in the ice phase and the PEB.

2 $$\mu_{1}^{\mathrm{ice}}=\mu_{1}^{\mathrm{brush}}$$

Before discussing further, it is important to note the nomenclature used in this paper. We break up the total volume into three parts: ϕ_1_ is the water volume fraction calculated using eq 3a, ϕ_2_ is the polymer plus nondissociated counterion volume calculated using eq 3b, and ϕ_*c*_ is the dissociated counterion volume fraction calculated using eq 3c. However, in our experiment, we assume that we are measuring the polymer volume fraction plus all ions ( $\overset{®}{\phi_{2}\;}$ , eq 3d). It is therefore important to note that all theoretical polymer volume fractions (ϕ_2_, “pure polymer volume fraction”) were converted to experimental polymer volume fractions ( $\overset{®}{\phi_{2}\;}$ , “polymer volume fraction”) and plotted accordingly.

$$\{\begin{array}{ll} \phi_{1}=\frac{v_{1}}{v_{1}+v_{2}+v_{\mathrm{ndc}}+v_{\mathrm{dc}}} & \left(3a\right)\\ \phi_{2}=\frac{v_{2}+v_{\mathrm{ndc}}}{v_{1}+v_{2}+v_{\mathrm{ndc}}+v_{\mathrm{dc}}} & \left(3b\right)\\ \phi_{c}=\frac{v_{\mathrm{dc}}}{v_{1}+v_{2}+v_{\mathrm{ndc}}+v_{\mathrm{dc}}} & \left(3c\right)\\ \overset{®}{\phi_{2}\;}=\frac{v_{2}+v_{\mathrm{dc}}+v_{\mathrm{ndc}}}{v_{1}+v_{2}+v_{\mathrm{ndc}}+v_{\mathrm{dc}}} & \left(3d\right) \end{array}$$

Equations (3a), (3b), (3c), and (3d) provide simplified examples of how volume fractions were determined (the full equations can be found in the SI). Here, *v*_1_, *v*_2_, *v*_ndc_, and *v*_dc_ are the volumes of water, polymer, volume of nondissociated counterions, and dissociated counterions, respectively.

Based on the formulation discussed in our previous publication on PE solution,^24^ we can write the expression for the chemical potential of water within the brush as shown in eq 4. In the original derivation of free energy, Muthukumar^35^ assumed the molar volume of the counterion and water to be the same size as the polymer repeat unit. Here, we account for differences in the molar volumes of the polymer repeat unit, water, and counterions.

4 $$\frac{\mu_{1}^{\mathrm{brush}}}{\mathrm{RT}}=\ln\left(1-\phi_{2}\left(1+\frac{\overset{®}{\alpha_{1}\;}\overset{®}{V_{c}\;}}{\kappa }\right)\right)+\phi_{2}\left(1+\frac{\overset{®}{\alpha_{1}\;}}{\kappa }\left(\overset{®}{V_{c}\;}-1\right)-\frac{1}{\kappa N}\right)+\frac{\alpha \phi_{2}^{2}}{\mathrm{RT}}+\frac{3\sigma^{2}a^{4}}{\phi_{2}}+\frac{\mu_{1,o,l}\left(T\right)}{\mathrm{RT}}$$

$$\{\begin{array}{ll} \overset{®}{\alpha_{1}\;}=\alpha_{1}\frac{Z_{2}}{Z_{c}} & \left(5a\right)\\ \overset{®}{V_{c}\;}=\frac{V_{c}}{V_{1}} & \left(5b\right)\\ \overset{®}{V_{\mathrm{ru}}\;}=\frac{M_{o}}{\rho \;*V_{1}} & \left(5c\right)\\ \kappa =\overset{®}{V_{\mathrm{ru}}\;}+\left(1-\alpha_{1}\right)\overset{®}{V_{c}\;}\frac{Z_{2}}{Z_{c}} & \left(5d\right) \end{array}$$

Where α_1_ is the effective degree of ionization, *Z*_2_ and *Z*_*c*_ are the valency of the polymer and counterion, respectively.^35–38^ *V*_1_ is the molar volume of water, taken as 18 $\frac{{\mathrm{cm}}^{3}}{\mathrm{mol}}$ , while *V*_*c*_ is the molar volume of the counterion; here, we assume *V*_*c*_ = *V*_1_. For PMETA, *V*_ru_ = 8.66 calculated using the monomer mass, *M*_*o*_ = 172.25 g/mol, and the density of the PE, ρ = 1.105 g/cm^3^. Here, N is the number of repeat units in the polymer chain. Since the molecular weight of PMETA is very high, we can take $\frac{1}{\kappa N}=0$ . In this formulation, we assumed the volume fraction of polymer and water within the brush are not a function of brush thickness (Alexander–de Gennes brush model). This simple model is consistent with the assumptions used to analyze the IR data. For clarity, we note that the parameters denoted as α_1_ and $\overset{®}{\alpha_{1}\;}$ in this work correspond to α and α_1_, respectively, in Muthukumar’s notation; α is the interaction energy term which is generally expressed as $\alpha =\epsilon_{1,2}-\frac{1}{2}\left(\epsilon_{1,1}+\epsilon_{2,2}\right)$ . Because there are three separate interaction energies (polymer, water, and counterion), in the derivation of the chemical potential term, we have combined all the three terms with a single effective term represented as α. In eq 4, we incorporated a term related to the chemical potential due to the brush, and σ is the grafting density in units of chains/unit area. The term “*a*” is defined as follows:

6 $$a^{2}=\frac{M_{o}}{N_{A}\rho h}$$

where *N*_*A*_ = 6.023 × 10^23^ mol^–1^ is Avogadro’s number; *h* is the monomer length in the units of dimension, and it is usually taken to be 0.25 nm.

Since ice is a pure phase, we can write the chemical potential of water in the ice phase as follows:

7 $$\mu_{1}^{\mathrm{ice}}=\mu_{1,o,c}\left(T\right)$$

The differences in chemical potential for pure ice and water can be expressed using the latent heat of fusion.

8 $$\frac{\left(\mu_{1,o,c}\left(T\right)-\mu_{1,o,l}\left(T\right)\right)}{\mathrm{RT}}=-\int_{T_{1}^{o}}^{T}\frac{\Delta_{f}h_{1}^{o}}{RT^{2}}dT$$

By rearranging eqs ([Disp-formula eq2]), ([Disp-formula eq4]), and ([Disp-formula eq8]) and by taking $\overset{®}{V_{c}\;}=1$ , we can formulate an equation to utilize in determining the volume fractions:

9 $$\ln\left(1-\phi_{2}(1+\frac{\overset{®}{\alpha_{1}\;}}{\kappa })\right)+\phi_{2}+\frac{\alpha \phi_{2}^{2}}{\mathrm{RT}}+\frac{3\sigma^{2}a^{4}}{\phi_{2}}=-\int_{T_{1}^{o}}^{T}\frac{\Delta_{f}h_{1}^{o}}{RT^{2}}dT$$

In prior studies,^24,39^ we showed how to determine the integral in eq 9 using the temperature dependence of the heat capacities of ice and water. It is important to note that when α_1_ = 0, the equation returns to a normal uncharged system.

The value of α is reported as −500 $\frac{J}{\mathrm{mol}}$ for similar PE (based on the reported value of χ = −0.2 and α = *χ RT*); here we only consider enthalpic contributions from short-range interactions and neglect long-range interactions - see Figure S7 for more details.^40^ At *T* = *T*_1_^o^, the integral is zero, and we are in equilibrium with water.

The fitting is achieved by assuming α_1_ = 1 for each case and allowing only σ to vary. To achieve the best possible fit, we fit all three counterions at the same time using eq 9 to achieve σ = 2.7 × 10^13^ $\frac{\mathrm{molecules}}{{\mathrm{cm}}^{2}}$ .

## Results and Discussion

### Controlling Probe Depth

We first show that by controlling the thickness of the brush layer, we can probe inside the brush layer, and we find that the penetration of the evanescent wave is shorter than the thickness of the swollen brush layer. The IR beam evanescent wave penetration depth is 200–500 nm depending on wavelength.^41,42^ Figure 1 presents the ATR-IR spectra of PMETAC brushes with two different thicknesses over a temperature range from 0 to −60 °C in the heating cycle, within the spectral window of 1500–4000 cm^–1^. Several distinct vibrational modes are observed: the water bending mode at 1610–1675 cm^–1^ (highlighted in red), the carbonyl stretching mode at 1675–1750 cm^–1^ (green), methyl and methylene stretching vibrations at 2800–3000 cm^–1^, O–H stretching vibrations corresponding to liquid water at 3200–3650 cm^–1^ (purple and black), and an ice-associated OH stretch appearing below 3200 cm^–1^ (blue). The spectrum collected at 0 °C reflects the state after melting, representing the vibrational signature of liquid water confined within the tethered polymer brush network.

**Figure 1:**
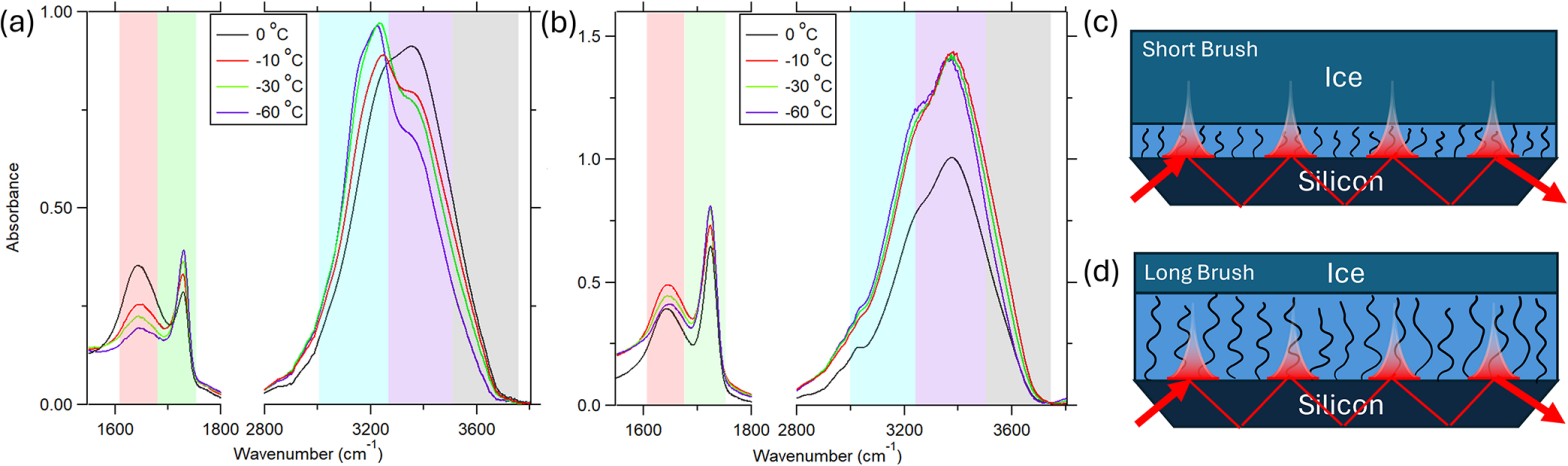
IR spectra in the heating cycle of short (a) and long (b) PMETAC brushes in the wavenumber range of 1500–4000 cm^–1^ at different temperatures: 0 °C (black), −10 °C (red), −30 °C (green) and −60 °C (purple). (c, d) Diagram of the IR cell showing the evanescent wave (red fading line) of the penetrating IR beam. The short brush (c) is shorter than the IR penetration depth, and water/ice signals from outside the brush are possible. The long brush (d) is longer than the penetration depth; as a result, all of the signals are generated from inside the brush network.

For the thicker brush (Figure 1b), minimal changes are observed in the water stretching region as the temperature decreases, indicating the persistence of nonfrozen liquid water within the brush network down to −60 °C. The small drop in OH stretching region in Figure 1b is not expected due to experimental conditions remaining constant. Repeated measurements indicate that sudden variations in absolute absorbance are not reproducible. Accordingly, we emphasize absorbance ratios, which provide a more robust and reliable metric than absolute absorbance. In contrast, for the short brush (Figure 1a), ice-related features become increasingly prominent at −10, −30, and −60 °C, as evidenced by the appearance of sharper ice-associated OH stretching bands. This differential behavior suggests that in thinner brushes, water external to the brush network undergoes freezing, while water confined within the thicker brush remains in the liquid state. Figure S5 gives a better comparison between the spectra for the short brush, the long brush, and pure water at low temperatures.

A schematic representation of this phenomenon is provided in Figure 1c. The solid red line represents the incident and reflected infrared beam traveling through the ATR crystal, while the fading red line indicates the evanescent field penetrating into the brush layer. In the case of the short brush, the penetration depth of the evanescent wave extends beyond the polymer layer, thereby sampling both the confined water within the brush and the external ice layer; this results in IR spectra that contain strong ice signatures. Conversely, for the 340 nm brush after swelling, the evanescent field remains predominantly confined within the brush layer, yielding spectra largely free of ice-like features and confirming the presence of supercooled liquid water within the brush network at subzero temperatures.

Figure 2a,b represent the ATR-IR spectra for the 304 ± 26 nm PMETA-Cl^–^ (blue), 324 ± 2 nm PMETA-I^–^ (light blue), and 296 ± 22 nm PMETA-SO_4_^2–^ (orange) brushes at temperatures of 0, −30, and −60 °C in the heating cycle for the 1500–1800 cm^–1^ (a) and 2800–3800 cm^–1^ (b) regions. As can be noticed in Figure 2a, the water bending mode at 1610–1675 cm^–1^ shows negligible change over the temperature range; however, the carbonyl stretching mode at 1675–1750 cm^–1^ shows small increases in intensity with decreasing temperature. This finding is attributed to changes in the polymer concentration within the IR penetration depth because of water leaving the polymer brush network to join the ice phase above. In the water stretching regions (Figure 2b), we observe minimal changes as the temperature decreases, supported by the idea that we are only probing within the brush layer (as illustrated in Figure 1c).

**Figure 2:**
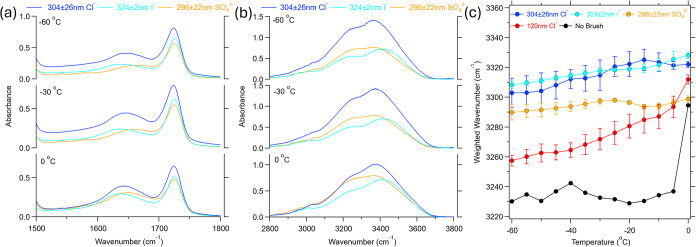
IR spectra of 304 ± 26 nm PMETA-Cl^–^ (blue), 324 ± 2 nm PMETA-I^–^ (light blue), and 296 ± 22 nm PMETA-SO_4_^2–^ (orange) brushes at temperatures of 0, −30, and −60 °C in the heating cycle for the 1500–1800 cm^–1^ (a) and 2800–3800 cm^–1^ (b) regions. (c) Calculated weighted peak location for the water stretching region in 120 nm (red) and 340 nm (blue) PMETAC brushes in the range of 0 to −60 °C. The calculation of the Weighted Average is taken from eq 1. The black line is a sample of blank silicon in contact with water. Error bars are the standard deviation of three different experiments. For blank silicon we are only showing results from one experiment. All data is taken in the heating cycle.

Figure 2c shows the calculated weighted average of the water stretching region (3100–3600 cm^–1^), as described in the Experimental Section and eq 1. Because the OH stretching band of liquid water is centered near 3300 cm^–1^, whereas ice exhibits a red-shifted band at approximately 3100–3200 cm^–1^, progressive ice formation produces an overall shift toward lower wavenumbers. This behavior is clearly observed for the control sample without any polymer brush, which shows the largest total shift (65 cm^–1^) between 0 and −60 °C, consistent with a complete transition from liquid water to bulk ice. The 120 nm PMETA-Cl^–^ brush shows an intermediate shift (40 cm^–1^), reflecting partial sampling of ice outside the thin brush due to the evanescent probe depth. In contrast, thicker brushes—including the 304 ± 26 nm PMETA-Cl^–^, 324 ± 2 nm PMETA-I^–^, and 296 ± 22 nm PMETA-SO_4_^2–^ samples—exhibit much smaller shifts, on the order of 20 cm^–1^. The minimal temperature-induced changes for these thick brushes indicate that the probed water is largely confined within the brush network and remains predominantly liquid even at −60 °C. The slight residual decrease in the weighted average wavenumber at low temperatures may reflect subtle restructuring of strongly bound or highly coordinated water within the brush, rather than bulk ice formation.

These results are consistent with the mechanism illustrated in Figure 1c, where in the thinner brush, the evanescent wave samples both the brush layer and the surrounding frozen water, resulting in a larger contribution from the ice phase. In the thicker brush, where the penetration depth remains within the brush network, the IR signal primarily reflects the confined water that remains nonfrozen even at temperatures well below 0 °C.

### Quantifying Polymer Volume Fraction

Having established that the effective IR probing depth can be controlled by tuning the polymer brush thickness, we calculate the fraction of water originally present in the swollen brush that remains nonfrozen (we refer to this quantity as *fraction of nonfrozen water*) (Figure 3a) and how the polymer volume fraction (Figure 3b) changes with temperature. To achieve this, a calibration curve was generated as described in the Experimental Section (Figure S4). This calibration process assumes that the polymer volume fraction is uniform within the brush. Using this calibration, we fitted the experimental low-temperature ATR-IR spectra to extract the corresponding polymer volume fractions at each temperature, which can be used to calculate the fraction of nonfrozen water within the brush. Figure 3a shows the fraction of nonfrozen water for samples of 304 ± 26 nm PMETA-Cl^–^ (blue), 324 ± 2 nm PMETA-I^–^ (light blue), and 296 ± 22 nm PMETA-SO_4_^2–^ (orange) brushes at temperatures of 0 to −60 °C in the heating cycle.

**Figure 3:**
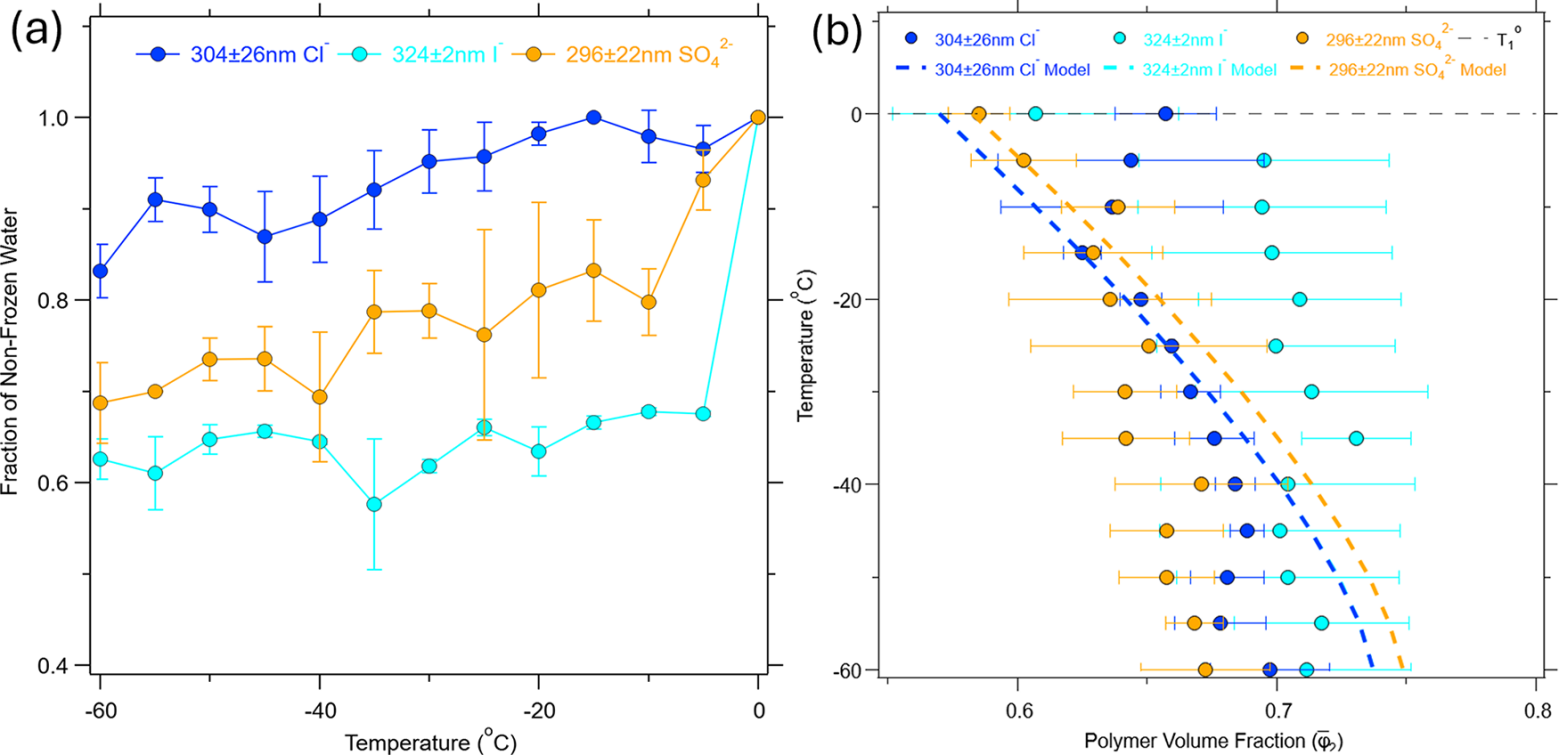
(a) Fraction of nonfrozen water within Cl^–^ (blue), I^–^ (light blue), and SO_4_^2–^ (orange) PMETA brushes in the temperature range of 0 to −60 °C in the heating cycle. (b) Polymer volume fraction as a function of temperature modeled for each counterion. The 324 ± 2 nm PMETA-I^–^ model (light blue) is plotted, but the line is overlapped by the line for the 304 ± 26 nm PMETA-Cl^–^ (blue) model.

At 0 °C, all the PMETA brushes exhibit a fraction of nonfrozen water of 1, this is when swollen brush is in contact with water. As the temperature is lowered to −60 °C, the nonfrozen fraction gradually decreases to 0.83 ± 0.03, 0.63 ± 0.02, and 0.69 ± 0.04 for Cl^–^, I^–^, SO_4_^2–^, respectively. This modest but measurable decrease is consistent with the expected deswelling behavior of the brush network due to the expulsion of freezable water during ice formation. The glass transition temperature (*T*_g_) of dry PMETA is reported to be approximately 120–160 °C. We anticipate that the *T*_g_ will be lower for PMETA in water and the nonfrozen water could be in a glassy state at low temperatures.^24^

Figure 3b plots the polymer volume fraction as a function temperature for 304 ± 26 nm PMETA-Cl^–^ (blue), 324 ± 2 nm PMETA-I^–^ (light blue), and 296 ± 22 nm PMETA-SO_4_^2–^ (orange) at temperatures of 0 to −60 °C in the heating cycle. At a temperature of 0 °C the starting polymer volume fractions are 0.66 ± 0.02, 0.61 ± 0.06, and 0.59 ± 0.01 and at −60 °C fractions of 0.70 ± 0.02, 0.71 ± 0.04, and 0.67 ± 0.02 for Cl^–^, I^–^, SO_4_^2–^ respectively. The model described in Experimental Section was used to fit the data using α_1_ = 1 and σ as the fitting parameters. The fitting parameter of σ = 2.7 × 10^13^ $\frac{\mathrm{molecules}}{{\mathrm{cm}}^{2}}$ was the best fit for the system when fitting all three counterions at the same time. Similar reports have found grafting densities ranging from 1.0 × 10^13^ to 5.0 × 10^13^ $\frac{\mathrm{molecules}}{{\mathrm{cm}}^{2}}$ .^43–47^ It is noted here that the model for the 324 ± 2 nm PMETA-I^–^ model (light blue) is plotted, but the line is overlapped by the line for the 304 ± 26 nm PMETA-Cl^–^ model (blue). To account for the uncertainty in the values of α, we have also varied this parameter and the corresponding value of the grafting density required to match the experimental data (Figure S7).

For applications such as anti-icing and cryopreservation, it is critical to maximize the amount of unfrozen water within the brush. Our theoretical model (eq 9) identifies that the grafting density predicts that lower grafting densities will promote a higher water uptake and a greater retention of nonfrozen water within the brush. This prediction of grafting density can be seen in Figure 4, where we varied the grafting densities of 1 × 10^13^ (red), 2.7 × 10^13^ (blue), 5 × 10^13^ (green), 7.5 × 10^13^ (light blue) $\frac{\mathrm{molecules}}{{\mathrm{cm}}^{2}}$ and predicted the polymer volume fraction as a function of temperature. The model prediction was based off of the experimental data of the 304 ± 26 nm PMETA-Cl^–^ model using an α_1_ = 1.

**Figure 4:**
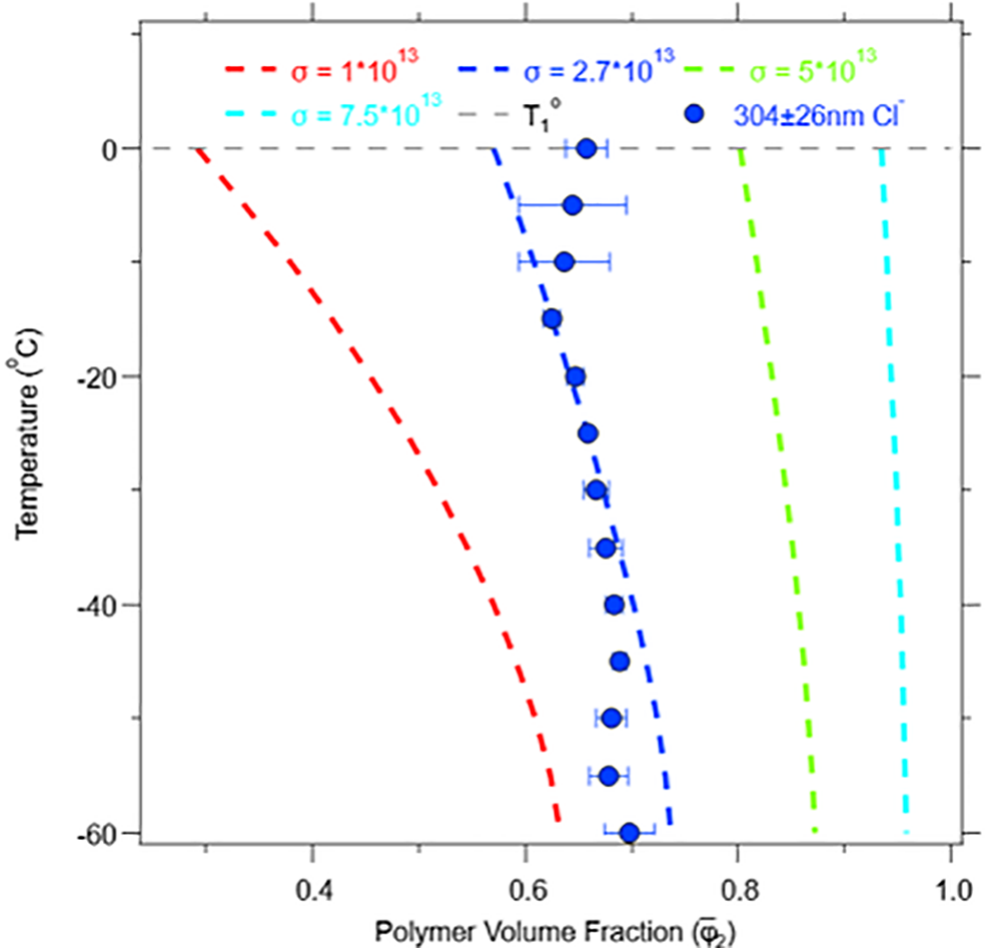
PMETA-Cl^–^ polymer volume fraction model as a function of grafting density (σ) ranging from 1 × 10^13^ (red), 2.7 × 10^13^ (blue), 5 × 10^13^ (green), and 7.5 × 10^13^ (light blue) $\frac{\mathrm{molecules}}{{\mathrm{cm}}^{2}}$ .

The ability to stabilize liquid water well below its bulk freezing point is reminiscent of antifreeze proteins and glycoproteins, which preserve nonfrozen water in biological systems. Beyond biology, this insight has implications for food preservation, where controlling water retention and ice formation is essential for texture and stability as well as for materials science, where confined water influences the performance of antifouling coatings and energy storage systems. In particular, the capacity of polyelectrolyte brushes to retain liquid water at subzero temperatures highlights their potential as synthetic analogs of antifreeze biomolecules and as functional components in next-generation anti-icing and cryo-lubricating coatings.

## Conclusion

In this study, we quantified the polymer volume fraction and the fraction of nonfrozen water present in swollen polyelectrolyte (PE) brushes. Using ATR-IR spectroscopy, we tracked how these quantities varied as a function of temperature, and overcome the limitations of bulk techniques and indirect methods, allowing selective, temperature-resolved probing of water within the polymer brush relative to surrounding ice. PE brushes are known to retain unfrozen water and reduce ice adhesion, and the quantification of such water is a broader question of importance in many biological systems. To this end, we constructed phase diagrams describing the coexistence of a positively charged PMETA brush with ice, using three different counterions of varying kosmotropic strength. These phase diagrams were modeled with a modified Flory–Huggins framework that incorporates both the chemical potential of water in the swollen brush and the extent of counterion dissociation.

Our findings provide direct evidence that PMETA brushes retain nearly 25-35 volume % of water in an unfrozen state at −60 °C. Whether these brushes become immobile at such low temperatures, potentially due to crossing the glass transition, remains unresolved and will be the subject of future investigation. Both the content of unfrozen water and the physical state of the PE brushes at subzero temperatures are critical considerations for the design of next-generation anti-icing and cryo-lubricating coatings.

## Supplementary Material



## Notes

Additional details about the IR and modeling data analysis (PDF)
