# Supplementary material for: Molecular Origins of Nonfrozen Water in Polyelectrolyte Brushes
Source: Langmuir. 2026 Mar 6;42(11):7754–61. doi: 10.1021/acs.langmuir.5c06138 (PMC13019683; doi:10.1021/acs.langmuir.5c06138)
Supplement: Supplementary file 1 [file la5c06138_si_001.pdf]

Supporting Information

Molecular Origins of Nonfrozen Water in Polyelectrolyte Brushes

George Mallinos, Md. Golam Kibria, Saveen Jayaweera, and Ali Dhinojwala*

School of Polymer Science and Engineering, The University of Akron, Akron OH 44325, USA

E-mail: ali4@uakron.edu

Contents

Number of pages: 12

Number of figures: 7

Number of equations: 7

List of Figures

S1 Comparison of the model to the spectra of dry PMETAC brush S4
S2 Optical constants for PMETAC S6
S3 Model predictions for IR spectra S7
S4 Calibration curves for the range of brush length S8
S5 Experimental IR comparison of the short PMETAC brush, the long PMETAC brush S8
S6 XPS results for counterion exchange S9

S7 Model fitting results S11

1. Calculation of polymer volume fraction within the swollen brush layer

In this section we describe how the ATR-IR spectra collected as a function of temperature were used to calculate polymer volume fraction within the brush. To make quantitative predictions we will model the data using optical modeling, ATR-IR spectra collected for a known thickness of polymer brush, and Bruggeman approximation to determine refractive indices of polymer brush swollen with different quantity of water. This combined approach allowed us to determine a calibration curve for ratio of absorbance of the carbonyl peak to the sum of both carbonyl and water bending peak.

The procedure described here is divided into various sections. In the first section, we use a three-layer optical model and a Gaussian oscillator model to represent complex dielectric constant, to determine the complex refractive indices of PMETAC polymer in the carbonyl region. The second step was to determine the complex dielectric constants for various volume fractions of PMETAC in water using Bruggeman approximation. The final step was to use a two-layer optical model to calculate the calibration curve where the y-axis is ratio of the absorbance in the carbonyl region to that of total absorbance of carbonyl and water bending region as a function of PMETAC volume fraction. This calibration can then be used to determine how polymer volume fraction changes with temperature for PMETAC brushes in the presence of three counterions.

A. Determination of refractive indices for PMETAC polymer

Figure S1a shows the ATR-IR spectrum for a 340nm thick PMETAC brush measured after heating the sample at 110 °C under nitrogen for 60 minutes. Under this condition, we did not observe a measurable water bending peak at 1643 cm^{-1}. The thickness of PMETAC brush was measured using a spectroscopic ellipsometer while blowing dry nitrogen over the sample.

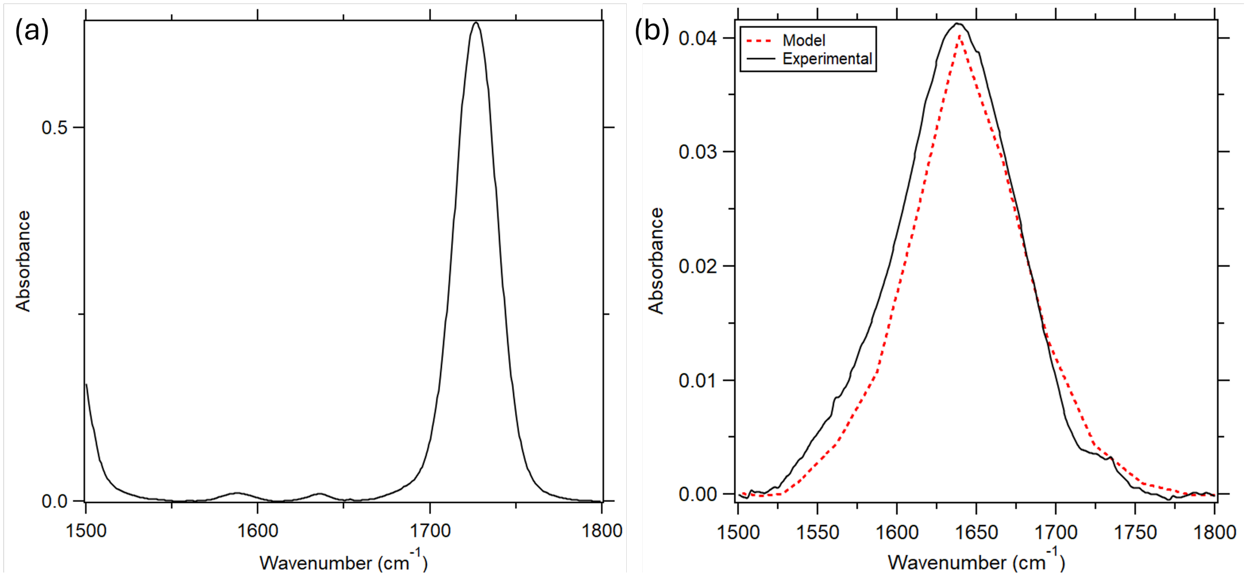

Figure S1: (**a**) Dry ATR-IR spectra of PMETAC brush in wavenumber region of 1500–1800 cm^{-1}. The brush was dried at 110 °C under nitrogen for 60 minutes. (**b**) Comparison of the model to the spectra of dry PMETAC brush, where the red dotted line indicates the model results and the black solid line indicates the experimentally collected results. The model was fitted to the experimental data using the Gaussian oscillator and best values from the fits were, $A_G = 0.301$, $E_c = 0.2141$ eV, and $B = 0.00368$ eV.

To model the absorbance we have used a three-layer optical model consisting of silicon, PMETAC, and air. The incident light of 45°at the silicon-PMETAC boundary was used for optical simulations. The reflected light was then used to calculate the absorbance. Since there are ten bounces of the IR along the silicon crystal, the total measured absorbance was divided by 10 to compare between the calculated absorbance.

To calculate the absorbance using the optical model, the only unknown is the refractive index of the PMETAC layer. Here, we have used a Gaussian oscillator to model the wavelength dependent refractive index. The imaginary part of the dielectric constant (ϵ_2) is given by,

$$\epsilon_2(E) = A_G(e^{\frac{-(E-E_c)^2}{\sigma^2}} - e^{\frac{-(E+E_c)^2}{\sigma^2}}) \tag{S1}$$

where

$$\sigma = \frac{B}{2\sqrt{ln2}} \tag{S2}$$

Here, A_G is the absorption amplitude, E_c is the oscillator energy, and B is the bandwidth of resonance. The real part of the dielectric function (ϵ_1) is calculated using the Kramers–Kronig integration of ϵ_2 and is given by

$$\epsilon_1(E) = \epsilon_\infty - \frac{2}{\pi} P \int_0^\infty \frac{\zeta \epsilon_2(\zeta)}{\zeta^2 - E^2} d\zeta \tag{S3}$$

where ϵ_∞ is the value of the real part of the dielectric function at higher energy; the value of ϵ_∞ is fixed at 2. The parameter P in **Equation (S3)** represents the Cauchy principal part of the integral, and ζ is the energy variable with respect to which the integration is performed. The refractive indices can be determined using $\epsilon(E) = (n(E))^2$, where ϵ and n are the complex dielectric index ($\epsilon(E) = \epsilon_1(E) - i\epsilon_2(E)$) and the complex refractive index ($n(E) = n_1(E) - ik(E)$), respectively. The wavelength of the light in units of nm can be written as $\lambda = 1239.84$ / E(eV). The results from the fits are shown in **Figure S1b** and the real and imaginary refractive indices by fitting the absorbance data are shown in **Figure S2**. The optimized parameters we obtained from the fits were $A_G = 0.301$, $E_c = 0.2141$ eV, and $B = 0.00368$ eV.

B. Bruggeman approximation to determine refractive indices of PMETAC in the presence of water

In this section we provide the model we have used to determine the complex refractive indices for PMETAC in water at various polymer volume fractions. The Bruggeman effective medium approximation (EMA) assumes that the effective dielectric constant of the combined system (t) is a function of the dielectric constants of the host molecules (ϵ_{host}) and guest (ϵ_{guest}) molecules.[1]

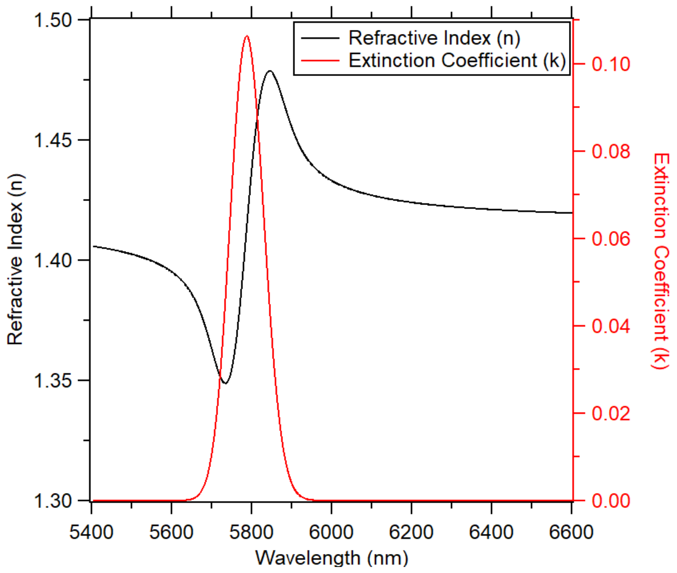

Figure S2: Optical constants for PMETAC. Refractive index (*black*) on the left axis and extinction coefficient (*red*) on the right axis in the wavelength region of 5400–6600 nm

$$\frac{\epsilon_{host} - \epsilon}{\epsilon_{host} - 2\epsilon}(1 - f) + \frac{\epsilon_{guest} - \epsilon}{\epsilon_{guest} + 2\epsilon} f = 0 \tag{S4}$$

where f is the volume fraction of the guest. Using **Equation (S4)** and the dielectric constants of the water (guest) and PMETAC (host), we can determine the dielectric constants of water containing different volume fractions of PMETAC.

C. Determination of the calibration curve

To calculate the absorbance spectra for various volume fraction of polymers, we have used a two-layer optical model. The first layer is silicon and the second layer is the PMETAC in water. We use an incident angle of 45 °and calculate the reflected light and determine the absorbance as a function of wavenumber. We then multiply this absorbance by 10 since we have 10 bounces of the IR beam along the length of the ATR crystal.

Figure S3 shows the absorbance calculated as a function of polymer volume fraction. As expected, the absorbance of carbonyl peak increases with increase in polymer volume fraction. The opposite trend is observed for the water bending peak. We calculate the area

of the 1727 cm^{-1} peak normalized by the area of the carbonyl and water bending peak at 1643 cm^{-1}. This area fraction is plotted as a function of polymer volume fraction in **Figure S4a**. To account for potential variation in the thickness determined using ellipsometer, we have used averages based on the lower, average, and upper bound for thicknesses of the PMETAC brush. This calibration was then used to calculate average polymer volume fraction as a function of temperature (**Figure S4b**).

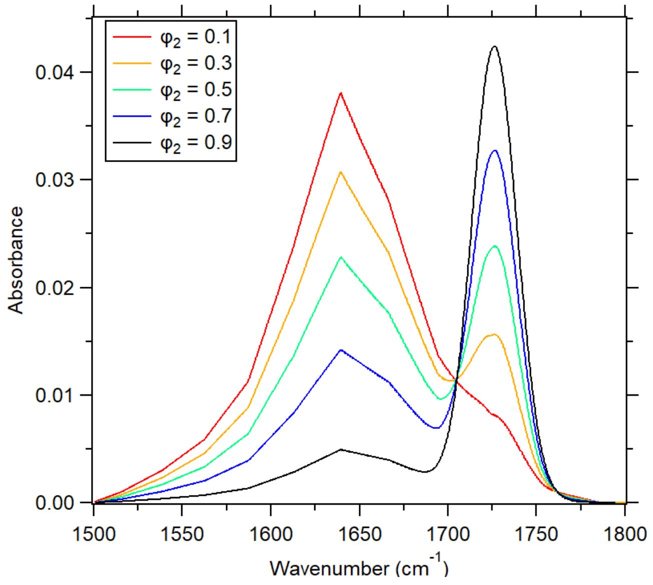

Figure S3: Model predictions for IR spectra at polymer volume fractions of 0.1, 0.3, 0.5, 0.7, and 0.9.

2. Experimental Data

Figure S5 shows a comparison of the spectra in the wavenumber range of 1500-4000 cm^{-1} for a short PMETAC brush, a long PMETAC brush, and a blank sample with no brush in water at temperatures of 0 °C (**a**) and –60 °C (**b**). It can be seen that the blank sample shows a strong peak in the 3100 cm^{-1} region in the –60 °C scan; this peak represents the formation of ice, which is not seen in the long brush system. In the short PMETAC brush, the same peak can be seen due to ice formation outside of the brush. This further illustrates the description of **Figure 1** in the main text.

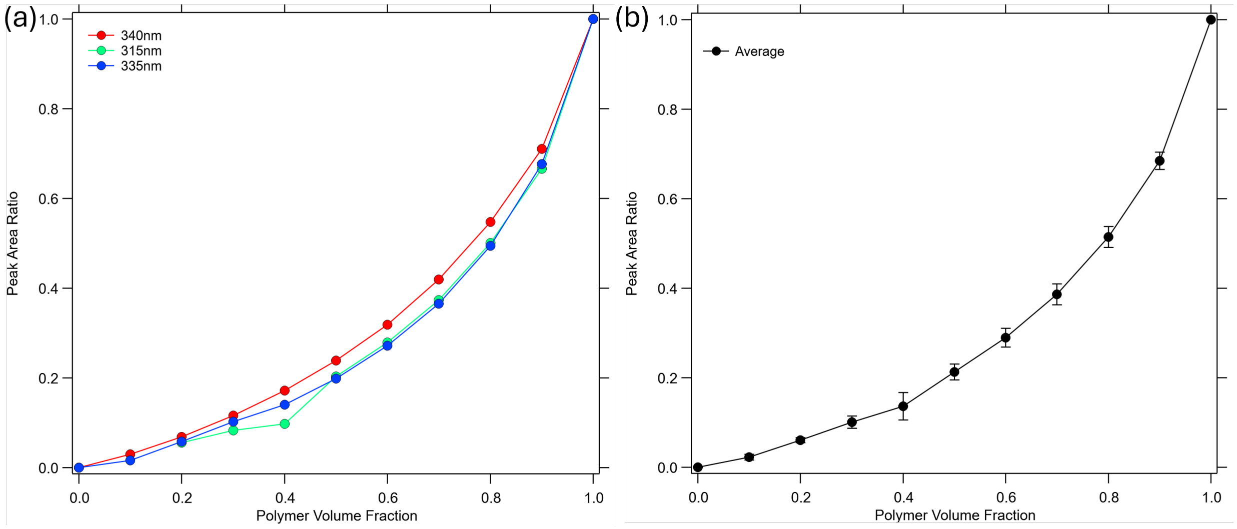

Figure S4: Calibration curves for the range of brush length (**a**) and the average with a fitted line to connect each point to the next (**b**).

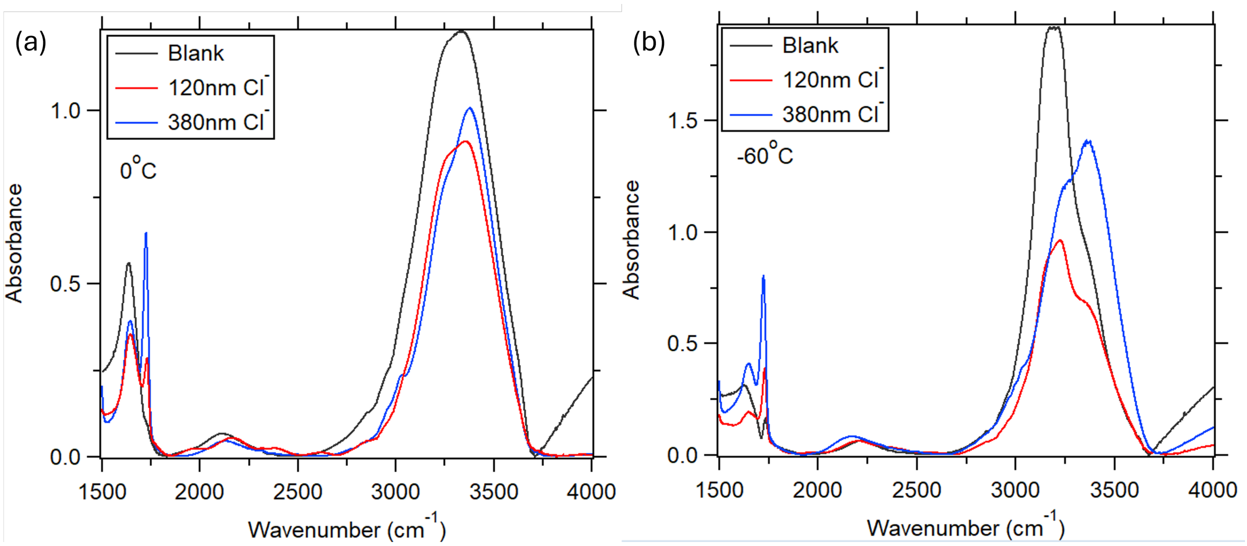

Figure S5: Experimental IR comparison of the short PMETAC brush (*red*), the long PMETAC brush (*blue*), and a blank sample with no brush (*black*) in the wavenumber range of 1500–4000 cm^{-1} for temperatures of 0 °C (**a**) and –60 °C (**b**)

3. X-ray Photoelectron Spectroscopy Analysis

The completeness of the ion exchange process was evaluated using X-ray photoelectron spectroscopy. Successful replacement of the original Cl^- counterions (**Figure S6a and 6b**) with SO_4^{2-} (**Figure S6c, and 6d**) was confirmed by the appearance of a distinct S2p signal at a binding energy of 168.6 eV, accompanied by a significant reduction in the Cl2p signal at 196.7 eV. The near-complete disappearance of the Cl2p peak indicates an efficient exchange of chloride ions. In addition, the Na 1s signal was detected at a negligible level ($<$0.1 atomic %), suggesting that free salt was effectively removed from within the polymer brushes.

(a)
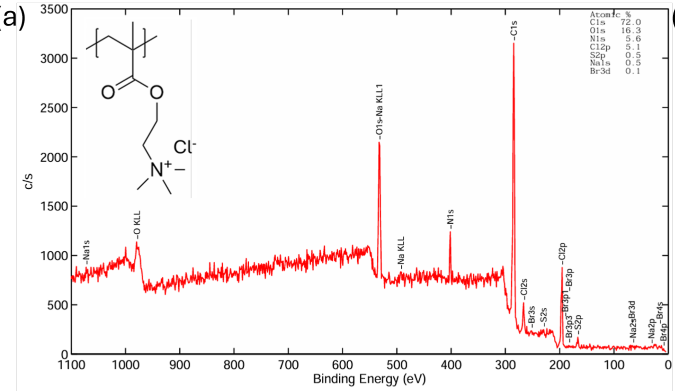

(b)

Atom	Count	Structure Atomic%	Avg XPS Atomic %
C	9	69.2	71.6 ± 0.46
O	2	15.4	17.0 ± 0.75
N	1	7.7	5.7 ± 0.32
Cl	1	7.7	5.0 ± 0.51
Br	0	<0.1	<0.1
Na	0	0	0.5 ± 0.07
S	0	0	0.4 ± 0.21

(c)
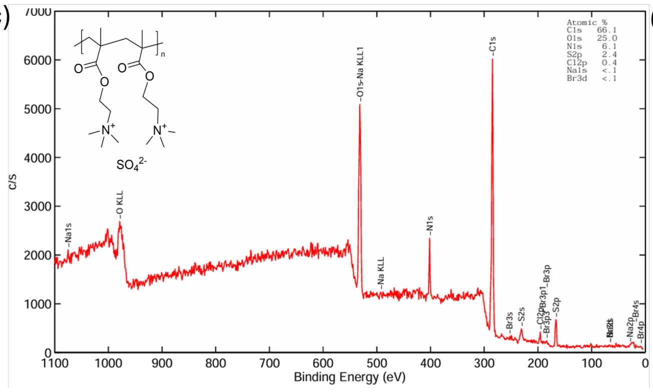

(d)

Atom	Count	Structure Atomic %	Avg XPS Atomic %
C	18	62.1	66.1
O	8	27.6	25
N	2	6.9	6.1
S	1	3.4	2.4
Na	0	0	<0.1
Cl	0	0	0.4
Br	<0.1	<0.1	<0.1

Figure S6: XPS results for counterion exchange of Cl^- (**a**,**b**) and SO_4^{2-} (**c**,**d**)

4. Thermodynamic Model

Equation (S5) is the free energy equation for the PMETAC solution when considering the three main components (water, polymer, and dissociated counterion).

$$\frac{F}{RT} = n_1 \ln \phi_1 + n_2 \ln \phi_2 + n_c \ln \phi_c + n_1\phi_2\chi_{12} + n_1\phi_c\chi_{1c} + n_2\phi_c\chi_{2c} \quad \text{(S5)}$$

where n_1, n_2, and n_c are the moles of water, polymer, and dissociated counterion respectively; ϕ_1, ϕ_2, and ϕ_c are the volume fraction of water, polymer+undissociated counterion, and dissociated counterion, respectively. Here, χ_{12}, χ_{1c}, and χ_{2c} are the interaction parameters for water–polymer, water–counterion, and polymer–counterion, respectively. The derivative of the free energy equation gives us the chemical potential of the solution, as expressed in **Equation (S6)**. This formulation of the chemical potential differs from those presented in previous reports,[2,3] where the counterion volume within the lattice model framework was assumed to be equivalent to that of the polymer repeat units. Here, we considered all three molar volumes to be non-equal to first determine the general equation for chemical potential:

$$\frac{\mu_1}{RT} = \ln(1 - \phi_2(1 + \frac{\overline{\alpha_1}\overline{V_c}}{\kappa})) + \phi_2(1 + \frac{\overline{\alpha_1}}{\kappa}(\overline{V_c} - 1) - \frac{1}{\kappa N}) + \frac{\alpha\phi_2^2}{RT} + \frac{3\sigma^2 a^4}{\phi_2} + \frac{\mu_{1,o,l}(T)}{RT} \quad \text{(S6)}$$

A. Volume Fractions

The volume fraction equations corresponding to **Equations (4a-4c)** in the main text are explained here. n_1 and n_2 are the moles of water and polymer, respectively, and α is the interaction energy term taking into account all the χ terms (outlined in the main text). The parameters α_1, $\overline{V_{ru}}$ $\overline{V_c}$, Z_2, and Z_c are explained in the main text. The term N is the number of repeat units in the polymer chain.

$$
\begin{cases}
\phi_1 = \dfrac{n_1}{n_2 N \overline{V_{ru}} + \overline{V}_c n_2 N \frac{Z_2}{Z_c} + n_1} & \text{(S7a)} \\
\phi_2 = \dfrac{n_2 N \overline{V_{ru}} + (1-\alpha_1)\overline{V}_c n_2 N \frac{Z_2}{Z_c}}{n_2 N \overline{V_{ru}} + \overline{V}_c n_2 N \frac{Z_2}{Z_c} + n_1} & \text{(S7b)} \\
\phi_c = \dfrac{\alpha_1 \overline{V}_c n_2 N \frac{Z_2}{Z_c}}{n_2 N \overline{V_{ru}} + \overline{V}_c n_2 N \frac{Z_2}{Z_c} + n_1} & \text{(S7c)} \\
\overline{\phi_2} = \dfrac{n_2 N \overline{V_{ru}} + \overline{V}_c n_2 N \frac{Z_2}{Z_c}}{n_2 N \overline{V_{ru}} + \overline{V}_c n_2 N \frac{Z_2}{Z_c} + n_1} & \text{(S7d)}
\end{cases}
$$

B. Enthalpic Term

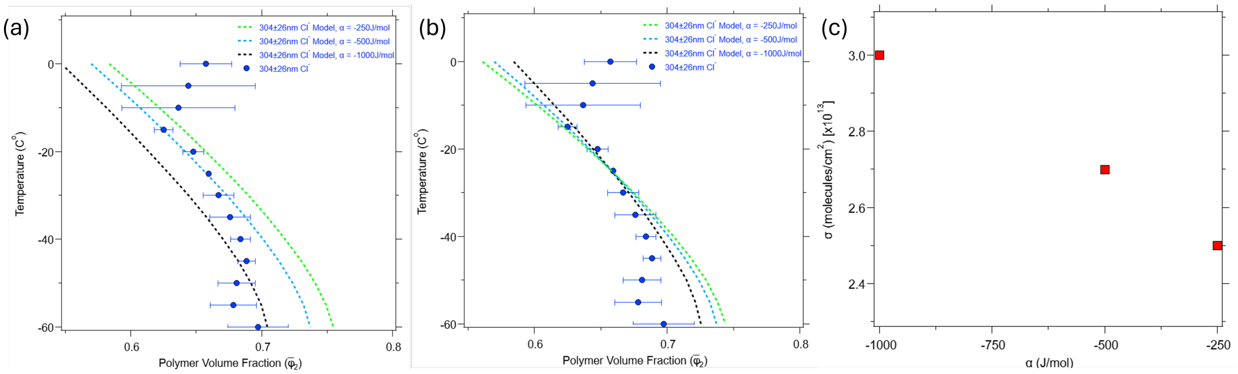

Figure S7: Fitting results for different alpha values (α = -250$\frac{J}{mol}$ (green), -500$\frac{J}{mol}$ (blue), and -1000$\frac{J}{mol}$ (black)) when holding $\sigma = 2.7 \times 10^{13}$ $\frac{molecules}{cm^2}$ (**a**) and when σ is allowed to vary (**b**). (**c**) is σ values for each α case

In the derived thermodynamic equation, the enthalpic term (α) accounts only for short-range interactions and neglects long-range electrostatic contributions. **Figure S7** illustrates the influence of incorporating these effects into our fitting. Because the value of χ for strong polyelectrolytes is not well established, we adopted $\chi = -0.2$, corresponding to the weak polyelectrolyte case of chitosan in water.[4] To examine the influence of both varying χ and including long-range interactions, we analyzed how changes in α affect the model fit under two conditions: (a) when σ is held constant at 2.7×10^{13} $\frac{molecules}{cm^2}$ (**Figure S7a**) and (b) when σ is allowed to vary (**Figure S7b**). The corresponding σ values for each α case are shown in **Figure S7c**. In both cases, these modifications produced only minor changes in the

overall thermodynamic behavior; however, further investigation is needed to fully elucidate the impact of long-range interactions on the system.

References

(1) Bruggeman, D. A. G. Berechnung verschiedener physikalischer Konstanten von heterogenen Substanzen. I. Dielektrizitätskonstanten und Leitfähigkeiten der Mischkörper aus isotropen Substanzen. *Annalen der Physik* **1935**, *416*, 636–664.

(2) Muthukumar, M. Phase diagram of polyelectrolyte solutions: Weak polymer effect. *Macromolecules* **2002**, *35*, 9142–9145.

(3) Mallinos, G.; Dhinojwala, A. Phase Diagram of Polyelectrolyte Solutions in Ice and Water. *J. Phys. Chem. B* **2025**, *129*, 3918–3927.

(4) Safronov, A.; Zubarev, A. Y. Flory–Huggins parameter of interaction in polyelectrolyle solutions of chitosan and its alkylated derivative. *Polymer* **2002**, *43*, 743–748.
